# Catheter and echocardiographic-focused management of primary pulmonary vein stenosis in premature infants: a single center 5-year experience

**DOI:** 10.1038/s41372-026-02611-2

**Published:** 2026-03-20

**Authors:** Keira Nassetta, Conor P. O’Halloran, Emily Hoyt, Matthew Cornicelli, Nicolas Porta, Jeremy Fox, Alan W. Nugent, Paul Tannous, Amanda Hauck

**Affiliations:** Department of Pediatrics, Division of Cardiology, Ann & Robert H. Lurie Children’s Hospital of Chicago, Chicago, IL USA

**Keywords:** Congenital heart defects, Medical imaging

## Abstract

**Background/Objectives:**

We describe the outcomes of pulmonary vein stenosis treatment in a cohort of premature infants using an assertive percutaneous management strategy with echocardiography as the primary method of surveillance.

**Subjects/Methods:**

This retrospective study included fifteen premature infants (median 24 weeks gestational age) that underwent pulmonary vein intervention from 2018-2023. The primary outcome was patient survival rate, and the secondary outcome was pulmonary vein preservation rate.

**Results:**

The estimated patient survival was 100% and 89% at 1 and 2 years after diagnosis. The pulmonary vein preservation rate was 88% and 83% at 1 and 2 years. Patients received a median of 4 echocardiograms between each cardiac catheterization and an average of 1 CT scan for every 1.5 years of observation.

**Conclusions:**

Pulmonary vein stenosis can be successfully managed with a percutaneous approach to pulmonary vein rehabilitation using echocardiography for surveillance, even in extremely premature infants.

## Introduction

Primary pulmonary vein stenosis (PPVS) describes neointimal proliferation of pulmonary veins in the absence of a preceding surgery or intervention involving the vessels [1]. PPVS is most often seen in premature infants, occurring in 1–5% of infants with bronchopulmonary dysplasia [2, 3]. Historically, outcomes for multivessel PPVS were poor [4–6]. In recent years, improved survival rates have been achieved using a catheterization-based approach with frequent reinterventions [7], with a one-year survival rate of 84%. The combination of frequent reinterventions and a mammalian target of rapamycin (mTOR) inhibitor produced a survival rate of 100% at a median of 2.2 years in one single-center study [8]. More recently, the use of an mTOR inhibitor was associated with less frequent interventions for pulmonary vein stenosis and a one-year survival rate of 93% from the time of medication initiation [9]. The ideal treatment strategy for PPVS continues to evolve, with centers adopting different approaches to intervention and disease monitoring.

The management of PPVS involves catheterization-based interventions (angioplasty and intravascular stenting), surgical venoplasty, and medical management. Various imaging modalities are used for inter-procedural screening including computed tomography (CT), magnetic resonance imaging (MRI), lung perfusion scan, and echocardiography [6, 10]. Frequent surveillance imaging is needed, with published pulmonary vein stenosis protocols recommending CT scans every 1–3 months in multivessel disease [8, 11]. Other centers have published missed diagnoses and false positives when using echocardiography for surveillance in comparison to CT [6, 10]. Echocardiography in PVS requires a comprehensive approach, as a diagnosis should not be made based solely on pulmonary vein gradients which do not correlate with gradients on catheterization [12]. The ideal surveillance strategy for PPVS is not known, and the risk of ionizing radiation or general anesthesia must be weighed against the risk of undiagnosed disease progression.

In 2018, our institution adopted an assertive percutaneous management strategy for PPVS using echocardiography as the primary method for surveillance between catheter interventions. We aim to demonstrate patient survival using a structured echocardiography-based surveillance approach to percutaneous pulmonary vein rehabilitation in premature infants, and the ability to develop a de novo pulmonary vein stenosis (PVS) program with survival rates similar to those described in the literature.

## Methods

We performed a retrospective chart review of all premature infants that underwent pulmonary vein intervention at the Ann & Robert H. Lurie Children’s Hospital of Chicago between August 2018 and November 2023. Prematurity was defined as a gestational age less than 37 weeks. Demographic data were obtained from the medical records, including gestational age, age at diagnosis and comorbidities. Medications for both pulmonary hypertension and pulmonary vein stenosis were reviewed and noted if they had been prescribed at any point during the study period. Imaging data were collected from available echocardiograms, CT scans, and lung perfusion scans (no MRIs were performed in this cohort). Data were collected from all available cardiac catheterizations, which characterized the location and severity of PPVS. Patients with an atrial septal defect and/or a patent ductus arteriosus were included in data collection. Patients with other congenital heart disease or heart transplant were excluded to isolate the effect of PVS in the absence of confounding congenital heart disease. Approval was obtained by the Institutional Review Board at Ann & Robert H. Lurie Children’s Hospital of Chicago.

**Fig. 1:**
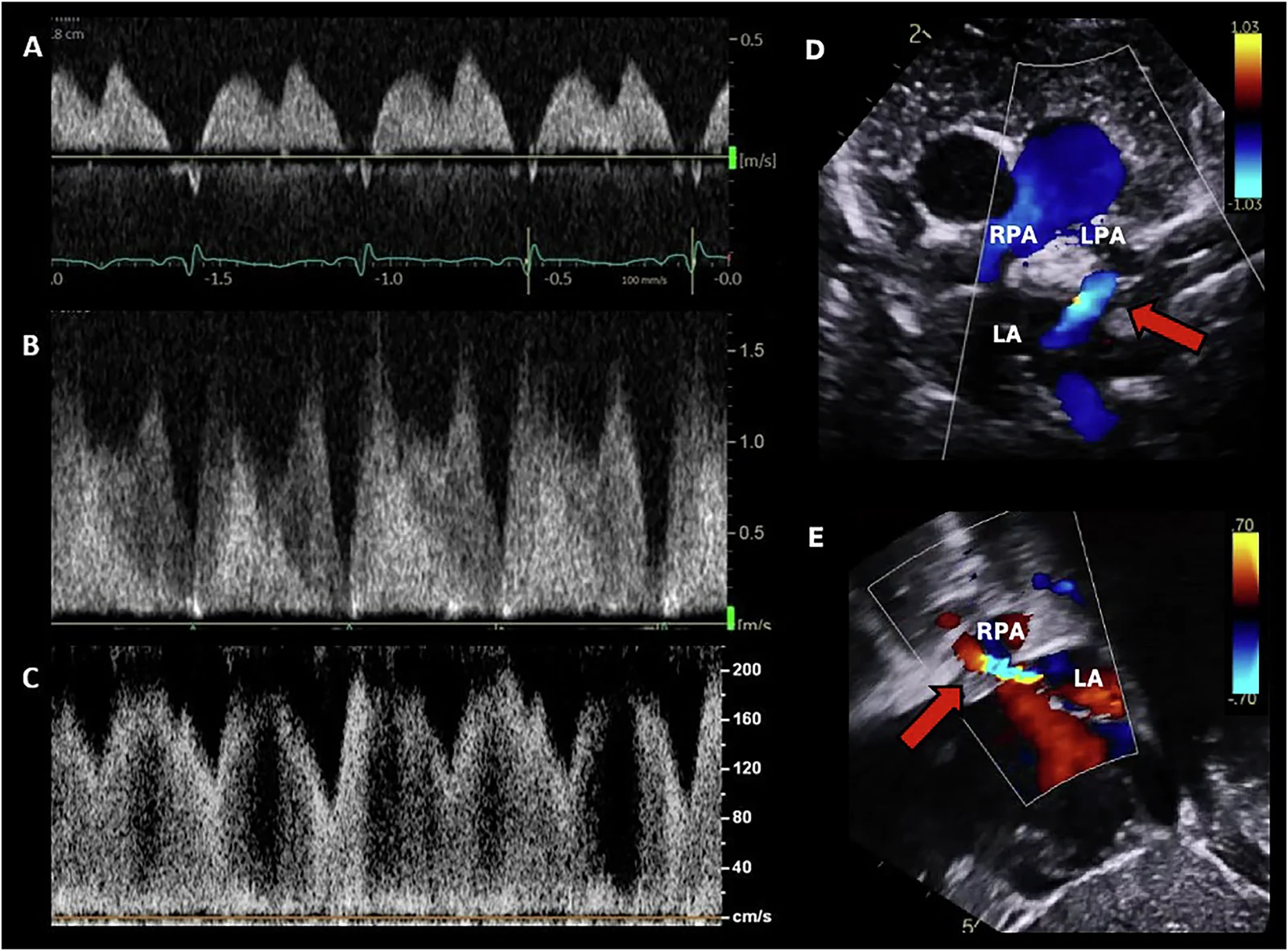
Echocardiographic findings in pulmonary vein stenosis. **A** Normal pulmonary vein Doppler. **B** Abnormal pulmonary vein Doppler that maintains a phasic pattern with return to baseline at end diastole but elevated peak velocity at 1.5 m/s and mean gradient of 4 mmHg . **C** Abnormal pulmonary vein Doppler without a normal phasic pattern, does not return to baseline at end diastole, elevated peak velocity at 2 m/s and mean gradient of 8 mmHg. **D** Suprasternal notch view with color demonstrating left upper pulmonary vein stenosis (arrow) with narrow color jet and flow acceleration as it crosses near the left pulmonary artery. **E** Subcostal sagittal view with color demonstrating right upper pulmonary vein stenosis (arrow) with narrow color jet and flow acceleration as it crosses the right pulmonary artery. RPA right pulmonary artery LPA left pulmonary artery RA right atrium LA left atrium.

Echocardiography was used as the primary surveillance method, initially performed every two to four weeks. Once the disease severity and rate of progression was understood, the frequency of echocardiograms was individualized. A standardized echocardiogram protocol was used. This protocol includes a qualitative assessment of each pulmonary vein size, both by two-dimensional appearance and by color Doppler. There is a focused evaluation on areas where the pulmonary veins have a higher risk of developing stenosis. The right upper pulmonary vein is interrogated as it crosses the right pulmonary artery, right bronchus and venoatrial junction. The left upper pulmonary vein is interrogated as it crosses the left bronchus proximal to and at the pulmonary vein-left atrium junction, and the left lower pulmonary vein is interrogated as it crosses the descending aorta. Pulmonary venous spectral Doppler tracings are obtained for all visualized veins and in any view where flow turbulence is identified. A normal pulmonary venous Doppler pattern was defined as a phasic pattern that returns to baseline at end diastole (less than 0.3 m/s), peak velocity less than 0.7 m/s and a mean gradient less than 2 mmHg [12]. Abnormal pulmonary vein Doppler patterns and abnormal color flow acceleration are displayed in Fig. 1. All new diagnoses identified by echocardiography were confirmed by CT angiography unless catheterization was planned for a different indication. If an affected pulmonary vein could not be visualized on an echocardiogram optimized for good technical quality, CT angiography was used to assess for disease progression. Repeat catheterization after initial intervention generally occurred three to six months later with input of the interventionalist’s understanding of the technical aspects of the case and guided by clinical and echocardiographic changes in patients. Echocardiographic indications for earlier intervention include signs of worsened pulmonary hypertension despite optimization of respiratory support, severely narrow or long-segment narrowing of pulmonary vein flow by 2D or color Doppler imaging concerning for evolving atresia, pulmonary vein gradient in an affected vein or unaffected vein increasing significantly, or clinical change with any of the above.

All patients with active PVS undergo monthly review by a dedicated multi-disciplinary team with representatives from non-invasive cardiac imaging (including echocardiography and CT/MRI), interventional cardiology, cardiac surgery, neonatology, and nursing. Ad hoc review occurs for any new diagnosis and following initial intervention if changes are identified that may warrant urgent reintervention. Plans for intervention and surveillance are created in accordance with group consensus. Percutaneous intervention is generally recommended in patients with multi-vessel PVS or patients with single vessel PVS who have pulmonary hypertension and/or persistent need for significant respiratory support (more than low flow nasal cannula). To provide consistency, all PPVS procedural plans undergo peer review with the interventional cardiology team and all echocardiograms were reviewed by a single cardiologist with expertise in PVS and pulmonary hypertension.

Data were entered into REDCap database by the research personnel. A vessel was considered atretic if it was not able to be visualized or recanalized at catheterization. Minor complication rate was defined as a transient change in patient status that may have required monitoring or a minor intervention and after which they returned to baseline [13]. Data are expressed as median (interquartile range), mean (standard deviation), or number (percentage) as appropriate. Analysis was performed using R Statistical Software (v4.1.2; R Core Team 2021). The primary outcome was patient survival rate. The secondary outcome was pulmonary vein preservation rate.

## Results

Between August 2018 and November 2023, 15 premature patients were treated for PPVS. The median gestational age was 24 weeks (23.5–25.5). The median age at diagnosis was six months (4.5–7). All patients had a diagnosis of bronchopulmonary dysplasia (11/15 severe BPD, 4/15 moderate BPD). An atrial septal defect or a patent ductus arteriosus was present in 14 (93%) patients. One patient had trisomy 21. One patient had a genetic defect in the EPHB4 gene. There were no other genetic syndromes. Demographic data are represented in Table 1. PPVS was diagnosed by echocardiogram in 14 (93%) patients. One patient was diagnosed via cardiac catheterization at the time of PDA closure.

**Table 1:** Demographic data.

Baseline characteristics	Overall
Number of patients	15
Age at PVS diagnosis (median (IQR))	6 months (4.5–7)
Estimated Gestational Age at birth (median (IQR))	24 weeks (23.5–25.5)
Small for Gestational Age (%)	7 (46.6)
Comorbidities	
BPD/CLD (%)	15 (100)
Moderate BPD (%)	4 (26.7)
Severe BPD (%)	11 (73.3)
ASD/PFO (%)	11 (73.3)
Any shunt (i.e., PDA or ASD) (%)	14 (93.3)
PDA s/p closure (%)	10 (66.7)
Age at PDA closure (mean (SD))	143.56 days (91.3)
Necrotizing enterocolitis (%)	9 (60)
Retinopathy of prematurity (%)	12 (80)
Genetic Syndrome (%)	2 (13.3)
Method of PVS Diagnosis	
Echocardiogram (%)	14 (93.3)
Catheterization (%)	1 (6.7)
Medications	
mTOR inhibitor (%)	4 (26.7)
Phosphodiesterase-5 inhibitor (%)	11 (73)
Endothelin Receptor Antagonist (%)	9 (60)
Prostacyclin (%)	3 (20)
Diuretics (%)	12 (80)

*PVS* pulmonary vein stenosis, *IQR* interquartile range, *BPD* bronchopulmonary dysplasia, *CLD* chronic lung disease, *ASD* atrial septal defect, *PFO* patent foramen ovale, *PDA* patent ductus arteriosus, *SD* standard deviation, *mTOR* mammalian target of rapamycin.

Eight patients (53%) had multivessel disease. There were 32 unique stenotic veins. The left upper pulmonary vein was the most frequently affected, accounting for 47% of stenotic vessels. This was followed by the right upper (25%), left lower (16%), and right middle pulmonary veins (12%). The right lower pulmonary vein was not affected in any patients.

Treatment involved 66 cardiac catheterizations. Patients received a median of two cardiac catheterizations per year. Stents were placed in 30 stenotic veins (94%). There were no procedural deaths or strokes in our population, and the most common complication was a blood transfusion (17%). Two patients underwent surgical intervention for pulmonary vein stenosis. One patient underwent surgical repair after stent placement caused compression of the left mainstem bronchus, and one patient underwent surgical repair for new ostial stenosis.

For the treatment of pulmonary hypertension, 11 patients were on a phosphodiesterase-5 inhibitor, nine patients were on an endothelin receptor antagonist, and three were treated with prostacyclin throughout their course. Pulmonary vasodilator therapies were used to treat pre-capillary pulmonary hypertension after pulmonary vein interventions to relieve post-capillary disease. For medical management of pulmonary vein stenosis, our center favored the mTOR inhibitor sirolimus. Four of the five patients with progressive disease were on sirolimus. No other patients were on sirolimus. Twelve patients were on diuretics at the time of intervention(s).

**Fig. 2:**
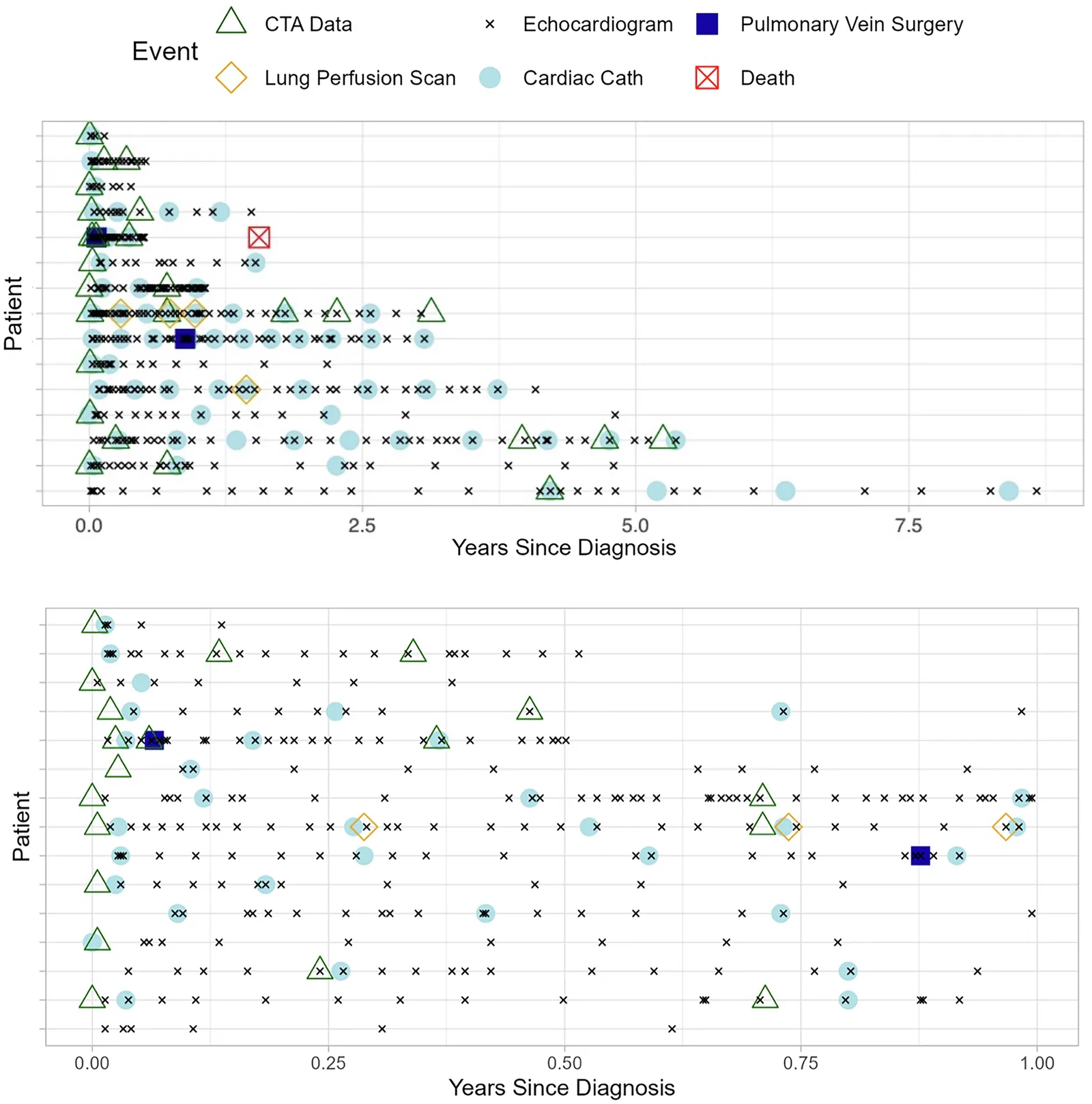
Frequency of surveillance imaging and interventions. Frequency of different modalities used for surveillance imaging and the number of cardiac catheterizations in our population.

Echocardiography was the primary means of surveillance between procedures. Patients received a median of four echocardiograms between each cardiac catheterization. Additionally, one CT scan was performed for every 1.5 years of observation on average. Three patients developed collateralization draining an affected lung segment and ultimately developed vein atresia without further attempts at canalizing the pulmonary vein. In one of these patients, the finding of pulmonary vein atresia was not expected at time of subsequent catheterization at 2.5 years of age. Only two patients received lung perfusion scans during the follow-up period. The number of interventions and frequency of surveillance imaging for each patient is represented in Fig. 2.

**Fig. 3:**
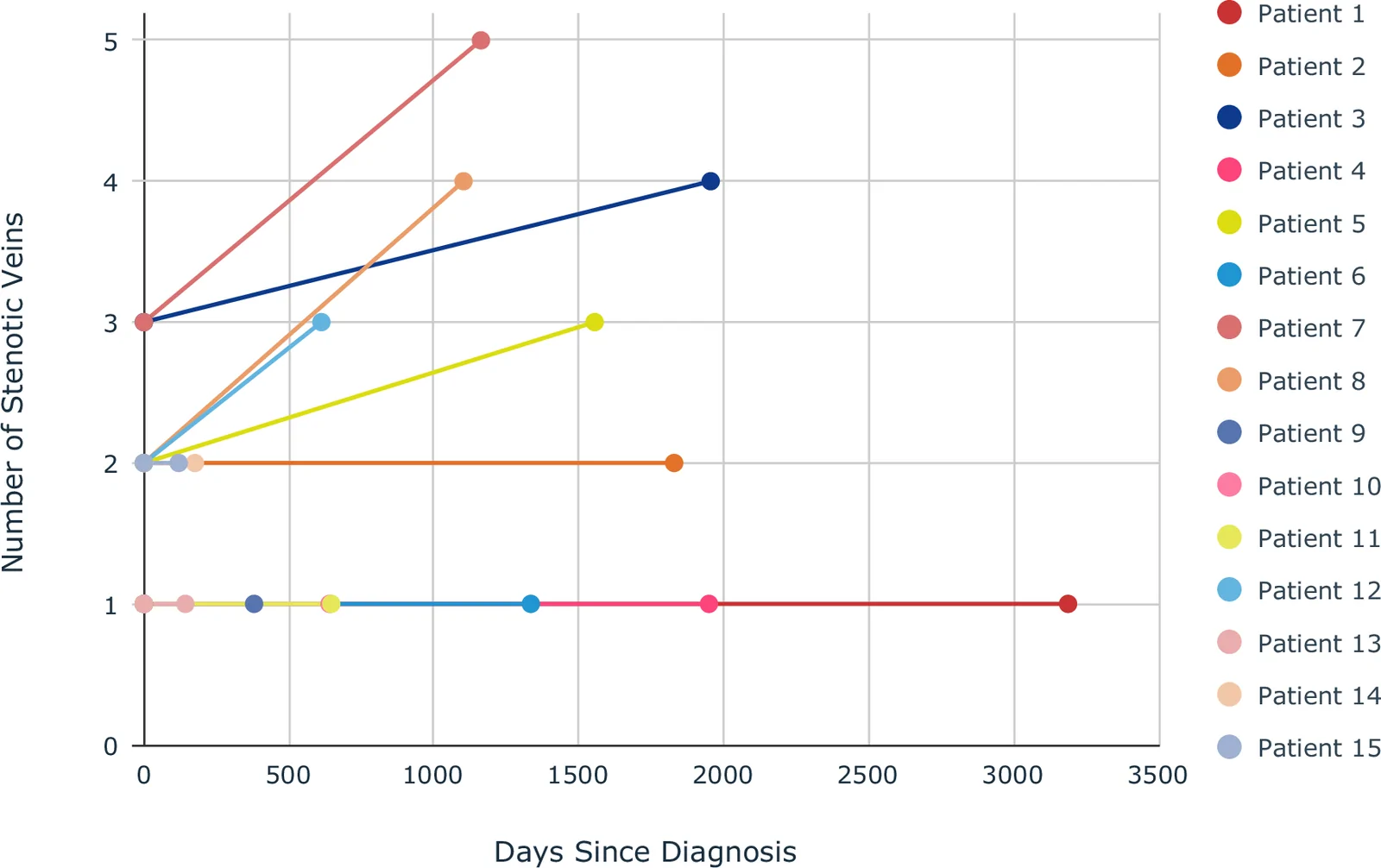
Progression of stenotic veins. Five patients developed stenosis of an additional pulmonary vein after their initial diagnosis. All five of these patients had more than one vein involved at the time of their initial diagnosis.

**Fig. 4:**
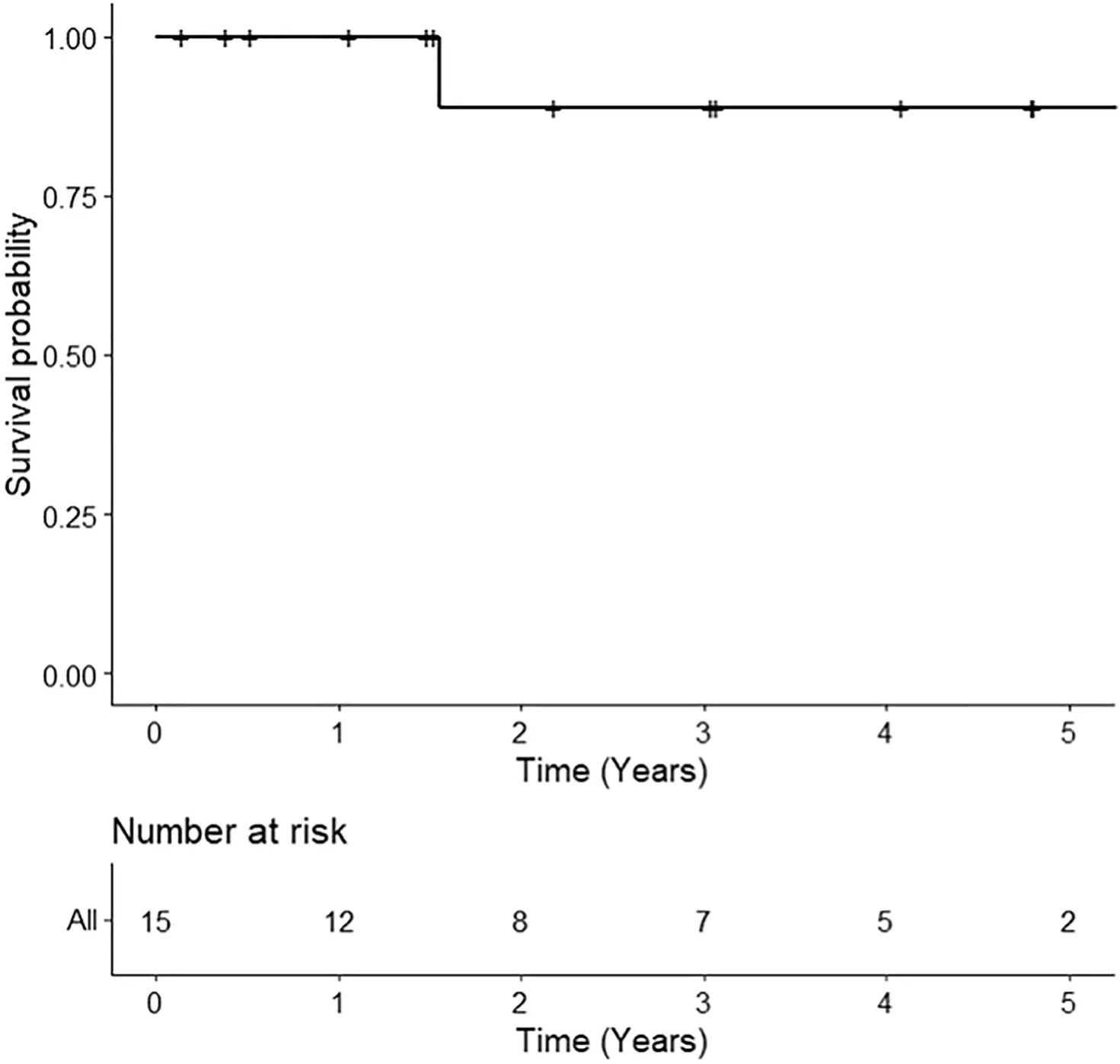
2-year survival rate. Kaplan-Meier for survival for all patients in the cohort. The estimated patient survival was 100% and 89% at 1 and 2 years after diagnosis.

**Fig. 5:**
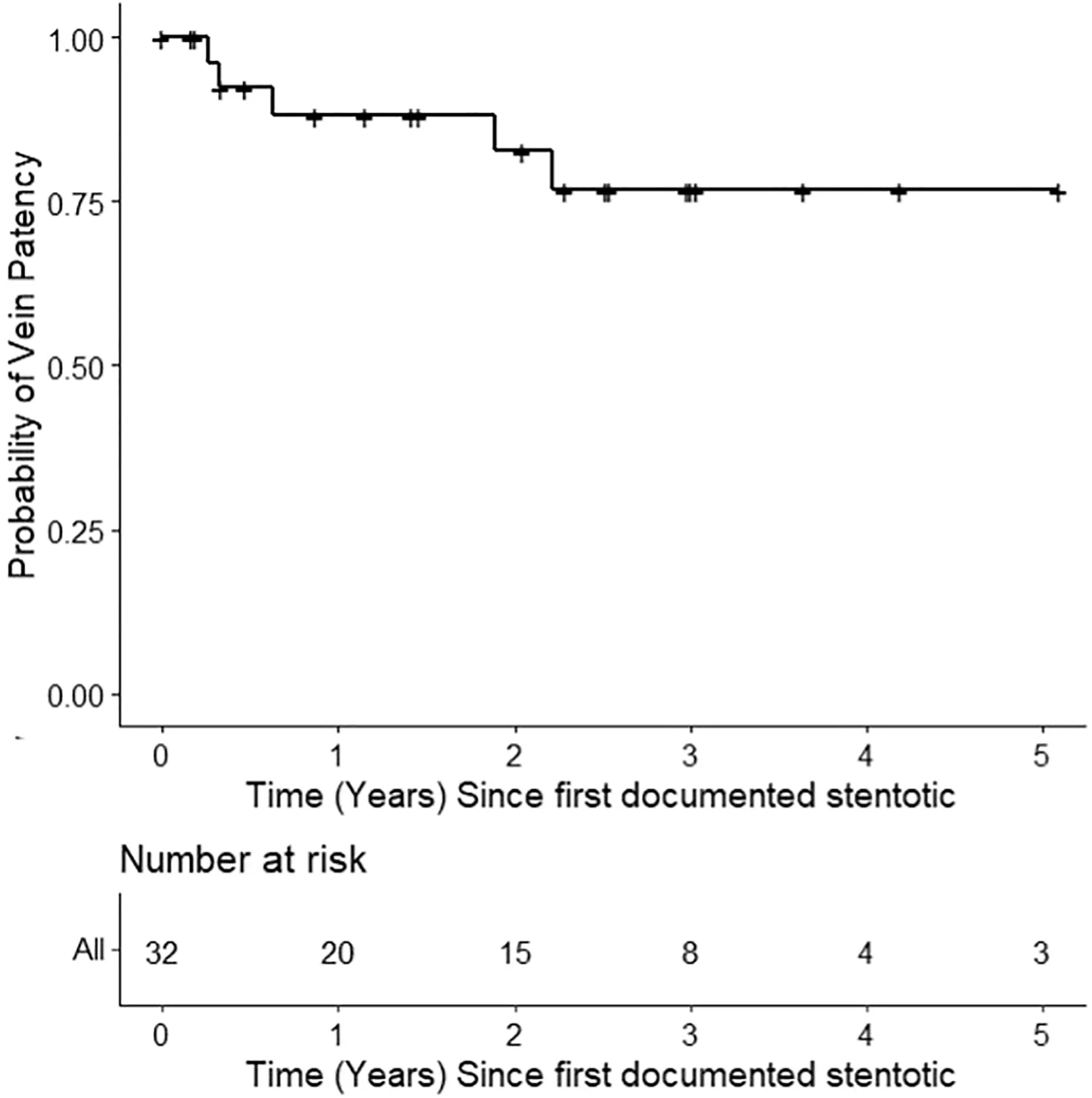
2-year vein preservation rate. Kaplan-Meier for number of preserved pulmonary veins for all patients in the cohort. The pulmonary vein preservation rate was 88% and 83% at 1 and 2 years after diagnosis.

Five patients developed de novo stenosis of additional veins after initial diagnosis (Fig. 3). All five patients with progressive disease had multivessel disease at the time of diagnosis. One patient died during the period of observation from bacteremia approximately one year after their last pulmonary vein intervention. An autopsy was performed and there was neither injury nor stenosis noted in the treated pulmonary veins. Using a Kaplan-Meier method with a median patient follow up from diagnosis of 2.2 years (IQR 1.3–4.4 years) estimated patient survival was 100% and 89% at 1 and 2 years after diagnosis (Fig. 4). With a median individual vein follow up from when first stenotic of 1.7 years (IQR 0.3–3.0 years), the pulmonary vein preservation rate was 88% and 83% at 1 and 2 years respectively (Fig. 5).

## Discussion

In this single center review of 15 premature infants with primary pulmonary vein stenosis, we demonstrate the success of an assertive percutaneous management strategy using echocardiography as the primary method for surveillance with minimal use of CT and perfusion imaging. Despite extreme prematurity, and high rates of significant comorbidities, our approach resulted in patient survival and vein preservation which are similar to contemporary published results. While published patient cohorts differ, Patel et al. demonstrated that among patients with at least moderate (i.e., multivessel) PVS, one and two-year survival rates were 74% and 64%, respectively [8]. These patients had a median gestational age of 34 weeks, which is older than our extremely premature population. In a heterogeneous population with PVS who were deemed candidates for mTOR inhibition, one and five-year survival rates from time of PVS diagnosis were 91% and 78%, respectively [9]. 14/45 (31%) of these patients were premature with gestational age less than 37 weeks [9]. Takajo et al. describe 56 patients with PVS with 36% dying at a median age of 10 months (IQR 4-24 months) [14]. Of the 56 patients, the median gestational age at birth was 37 weeks and only one was born very preterm (29 weeks) [14]. Due to our small sample size and the heterogeneous populations in the current literature, we were unable to perform a formal comparison with non-inferiority testing.

The primary novel component of our approach is the reliance on echocardiogram as the primary surveillance modality, with limited use of CT or perfusion imaging. The frequency of echocardiographic screening was highest in the six-month period after initial diagnosis and intervention (Fig. 2). At our institution, patients with severe pulmonary vein stenosis may receive echocardiograms as frequently as every 2 weeks, which is then spaced based on the trajectory of re-stenosis. An initial CT scan was performed in most patients to confirm the diagnosis, define the anatomy of the stenotic and adjacent veins, and to plan for interventions. Subsequent CT scans were only performed if the involved pulmonary veins could not definitively be seen by echocardiography on two successive studies due to technical limitations, or if there were other findings suggestive of worsening disease. CT scans were also incorporated when pulmonary vein interventions were spaced beyond one year to help guide timing of reintervention. Using this approach, patients received one CT scan for every one and a half years of observation, including the initial CT. A recent study evaluated the sensitivity and specificity of individual echo parameters in identifying pulmonary veins that require intervention, with qualitative vein narrowing and aliasing flow demonstrating high sensitivity (82% and 77%) and specificity (87% and 87%, respectively) for at-risk pulmonary veins [15]. Our PVS surveillance protocol relies similarly on these qualitative findings and emphasizes a multi-view assessment of anatomic at-risk areas for PVS. This provides a framework which is not currently described in the literature. A standardized protocol for echocardiographic surveillance can be adopted to reduce radiation exposure and transport of high-risk infants for imaging studies between interventions. As clinical indicators may be limited in this young population, frequent, protocolized echocardiograms can improve surveillance by establishing disease trajectory and informing intervention timing. For centers that may not have the resources to perform protocolized echocardiograms, other imaging modalities including CT scans and perfusion scans can be used for PVS diagnosis and surveillance. Multicenter collaboration with the consistent application of echocardiographic surveillance is necessary for the development of evidence-based screening guidelines that can be widely adopted.

As mentioned previously, only two of our patients underwent surgical intervention for PPVS. At our institution, there was a multidisciplinary consensus to favor the catheter-based management of PPVS given the risks of cardiopulmonary bypass in premature infants with severe bronchopulmonary dysplasia. As such, our program aggressively utilizes stenting for the treatment of PPVS. Stents can be dilated to account for somatic growth. When coronary stents are used in the upper pulmonary veins they can be post-dilated to account for growth throughout childhood (up to 6 mm). In contrast, coronary stents generally cannot achieve sufficient diameters for the lower pulmonary veins, and thus require fracturing. We therefore attempt to use bare metal stents for the lower pulmonary veins whenever technically feasible. Stent placement does not prohibit future surgical intervention, and patients may be better surgical candidates after initial palliation. Others have shown that patients remain at risk for recurrent PVS after surgical intervention, with one study demonstrating that approximately half of stents placed for PVS were placed after prior surgical intervention [16]. Long term data from multiple centers is necessary to elucidate future implications of stent placement, and to aid clinicians in decisions about interventional strategies.

Roughly half of our patients had multi-vessel disease at the time of diagnosis. Our patients with multivessel PPVS undergo repeat catheterizations as frequently as every 6-12 weeks. Repeat catheterization may be triggered by a change in clinical status, concern for worsening stenosis on echocardiography or at the request of the treating interventionalist. The frequent interventional catheterizations come with an acceptable risk profile. There were no procedural deaths or strokes in our patient population. Approximately one in five patients received a blood transfusion. The one fatality in our cohort occurred a year after their last vein intervention, and cause of death was multi-system organ failure with bacteremia. This patient was not on sirolimus. In our cohort, all patients with progressive disease had multivessel disease at the time of diagnosis. Multivessel disease at time of diagnosis should prompt clinicians to counsel families on the chronic nature of the disease with anticipated need for frequent screening and repeat interventions.

In a recent multi-center study of infants with bronchopulmonary dysplasia (BPD), the presence of an ASD was associated with the development of PVS, but a PDA was not [3]. Exposure to a left to right shunt has been shown to be a risk factor for the development of pulmonary vein stenosis in patients with trisomy 21 [17]. One of our patients had trisomy 21, and the majority (93%) of our patients had a left to right shunt. Eleven of our patients had an ASD, and 10 patients with a PDA underwent PDA device closure prior to PVS diagnosis. This observation supports the hypothesis that increased flow through the pulmonary veins leads to wall shear stress and neointimal proliferation [17, 18]. Independently, bronchopulmonary dysplasia is a well described risk factor for PPVS. All our patients had bronchopulmonary dysplasia, and the median gestational age was extremely premature at 24 weeks. Altogether, the extremely premature infant with bronchopulmonary dysplasia and a left to right shunt appears at higher risk for developing PPVS. Alternatively, it is possible that frequent echocardiograms performed in patients with cardiac shunt lesions leads to the diagnosis of PPVS, with cases of PPVS relatively underdiagnosed in infants without cardiac disease. However, all infants with BPD undergo monthly surveillance for pulmonary hypertension. These screening echocardiograms include the evaluation of all pulmonary veins [19, 20]. This supports the role of a cardiac shunt in the development of PVS.

There are limitations in this study. This is a retrospective chart review without randomization to intervention. This study only involved a single center in a large academic institution and is subject to referral bias. The sample size precludes determination of mortality risk based on number of affected veins. The small sample size reduces the power of our analysis, however this represents a substantial cohort for this disease process.

## Conclusion

Primary pulmonary vein stenosis can be successfully managed with a percutaneous approach to pulmonary vein rehabilitation using echocardiography for surveillance, even in extremely premature infants. A de novo pulmonary vein stenosis program can be developed with good outcomes. A targeted, multi-disciplinary approach is required for the care of these patients, including team members with expertise in interventional cardiology, cardiac imaging, neonatology, and pulmonary hypertension.

## Data Availability

Data presented in the manuscript will be made available to readers promptly upon request.
